# Comparative analysis of environmental standards to install a rooftop temperature monitoring station

**DOI:** 10.1038/s41598-022-27070-5

**Published:** 2022-12-27

**Authors:** Byeongtaek Kim, Sungeun Hwang, Youngtae Lee, Seungsook Shin, Kihoon Kim

**Affiliations:** 1 Observation Research Department, National Institute of Meteorological Sciences, 33 Seohobukro, Jeju, 63568 Republic of Korea; 2 Forecast Research Department, National Institute of Meteorological Sciences, 33 Seohobukro, Jeju, 63568 Republic of Korea

**Keywords:** Climate sciences, Environmental sciences

## Abstract

Urban climate influences economic activities and the health and safety of urban residents. Therefore, monitoring temperature in urban areas is important. However, owing to the lack of space for an appropriate observation site, an automatic weather station (AWS) was installed on a building rooftop. The rooftop installation can indicate temperature differences depending on the intensity of strong solar radiation and radiant heat of the building, and wind speed. Therefore, in this study, we aimed to provide observation standards for measuring rooftop temperature according to the optimal rooftop material and observation height. Specifically, an AWS was installed on the rooftop of the Gochang Standard Weather Station (GSWO), Jeollabuk-do Province, to observe the urban climate in South Korea and establish suitable weather standards. Different temperatures, optimum surface materials, and optimum heights for measuring the temperature at the rooftop of GSWO were investigated and compared over 1 year. The temperature recorded after installing a palm mat on the rooftop was more similar to that observed in the grassland. Furthermore, the installation height of the temperature sensor of 2.5–3.0 m for the palm mat and 3.5–4.0 m for concrete was found to be the optimal height for observing temperature at the rooftop.

## Introduction

The Korea Meteorological Administration (KMA) has installed and currently operates 635 automatic weather stations (AWS) and automated synoptic observation systems (ASOS) for weather observation. On-ground meteorological observations are represented by the general weather conditions near an observatory. Thus, observatories are not located in areas with tall buildings or with low visibility due to the presence of geographical barriers, such as mountains, forests, and lakes, but in open areas with widespread visibility^1^. However, rapid urbanization and severe climate change events have increased the intensity and frequency of heatwaves in major cities; consequently, the demand for accurate weather observation data has substantially increased^2–7^.

Meteorological observations can be used to develop and validate the accuracy of numerical weather predictions, microclimate analyses, and numerical prediction systems^8–12^, which in turn improves the accuracy of predicting floods, typhoons, and air pollution^13–15^. To this end, the KMA has installed an AWS in Seoul, Korea to increase the horizontal resolution of the observation network. Due to the lack of space for an appropriate observation site, the AWS was installed on a building rooftop. However, the rooftop installation can show temperature differences depending on the intensity of strong solar radiation, radiant heat of the building, and wind speed^16,17^. According to the World Meteorological Organization (WMO)^1^, the reference site for temperature measurement should be covered with vegetation of less than 10 cm. Therefore, minimizing the impact of artificial heat transmitted from the concrete floor of the roof and establishing installation standards are necessary to ensure that the temperature measurements are the same as those reflected by installations on land with grass patches (hereafter referred to as grasslands). Artificial heat transmission can be avoided by using either light colored rooftop packaging materials or reflective coatings to lower surface temperatures^18,19^. Evapotranspiration from plants and albedo changes on a green roof reduce the surface and near surface temperatures and decrease the average radiant temperature by 10.5 °C at noon when solar radiation is the strongest^20–23^. However, installing a green roof system is disadvantageous in terms of construction, maintenance, and removal costs and structural problems due to increased loads^24–26^.

According to the KMA standards for rooftop monitoring installations, thermometers should be located at least 1.2–2.0 m above the rooftop floor and installed in green areas to block solar radiation or on materials such as palm mats and grass, with low conductivity^27^. However, specific height and floor material criteria for accurate temperature measurements have not been provided. Several organizations have prescribed height guidelines for temperature observations; for example, the World Meteorological Organization^1^, American Association of State Climatologists^28^, and United States Environmental Protection Agency^29,30^ stated optimum installation heights as 1.25–2.0 m, 1.5–2.0 m, and 2.0 m, respectively. However, these guidelines are based on grassland standards and not rooftop standards. Studies have been conducted to compare rooftop and grassland temperatures to derive the optimal height for rooftop temperature observations^31–33^; however, these studies lacked sufficient sampling data and did not consider seasonal variations. A comparative analysis of the grassland temperature after installing artificial grass on a concrete rooftop revealed that the temperature measured in artificial grass was higher than that in the grassland.

This study aimed to determine the optimal material (concrete or palm mat) of rooftop surfaces for temperature observations by comparing the values with those measured at a standard observation site (grassland) of the Gochang Standard Weather Station (GSWO). Specifically, we analyzed seasonal temperature measurements to compare the air temperature differences between building rooftops and grasslands over 1 year (four seasons) from December 2020 to November 2021. Moreover, in this study, we derived the optimal installation heights of temperature sensors that are not affected by the cooling effect of wind and rainfall. Based on the experimental results, optimal installation heights for measuring temperature are suggested for each rooftop surface material and scientific and objective standards for the rooftop environment required to install thermometers for measurement are presented.

## Methods

### Study area

**Figure 1:**
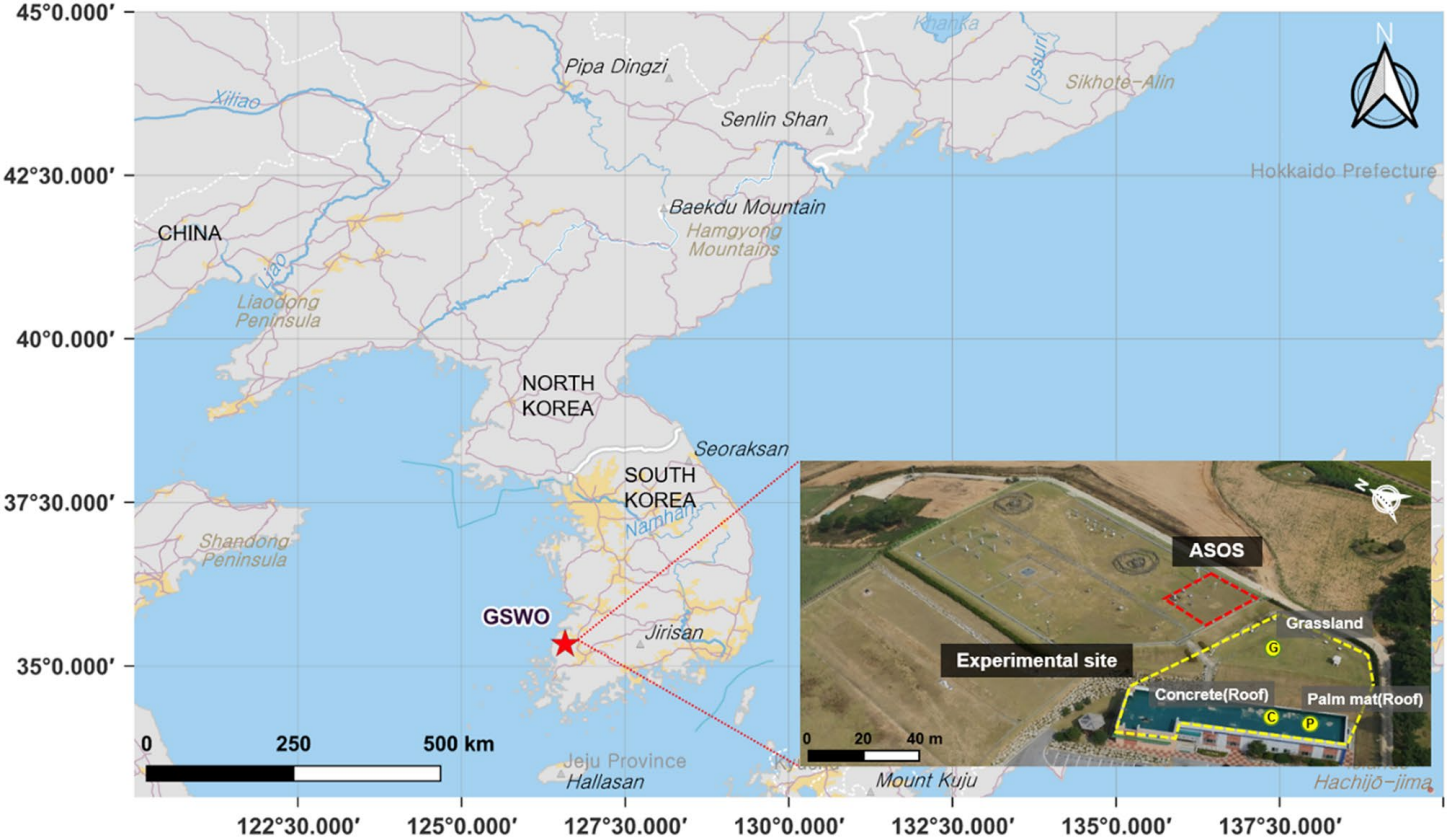
Location of the study area including ASOS and the experimental site (with grassland, concrete, and palm mat). This map was created with QGIS 3.26, https://www.qgis.org. Photograph by NIMS.

The study was conducted at GSWO (Fig. 1; 35° 20′ N, 126° 35′ E; altitude = 52.0 m), located in Gochang-gun, Jeollabuk-do, South Korea. GSWO has been designated and operational as a standard meteorological observatory since 2010; additionally, international standard observation facilities and ASOS are installed in GSWO^34^. GSWO is located in southwest Korea, which experiences temperatures higher than the daily average and low temperatures in Korea^35^. Therefore, its location is optimal to conduct comparative experiments to prepare standards for rooftop temperature observations and installation environments (Fig. 1).

### Experimental design

**Figure 2:**
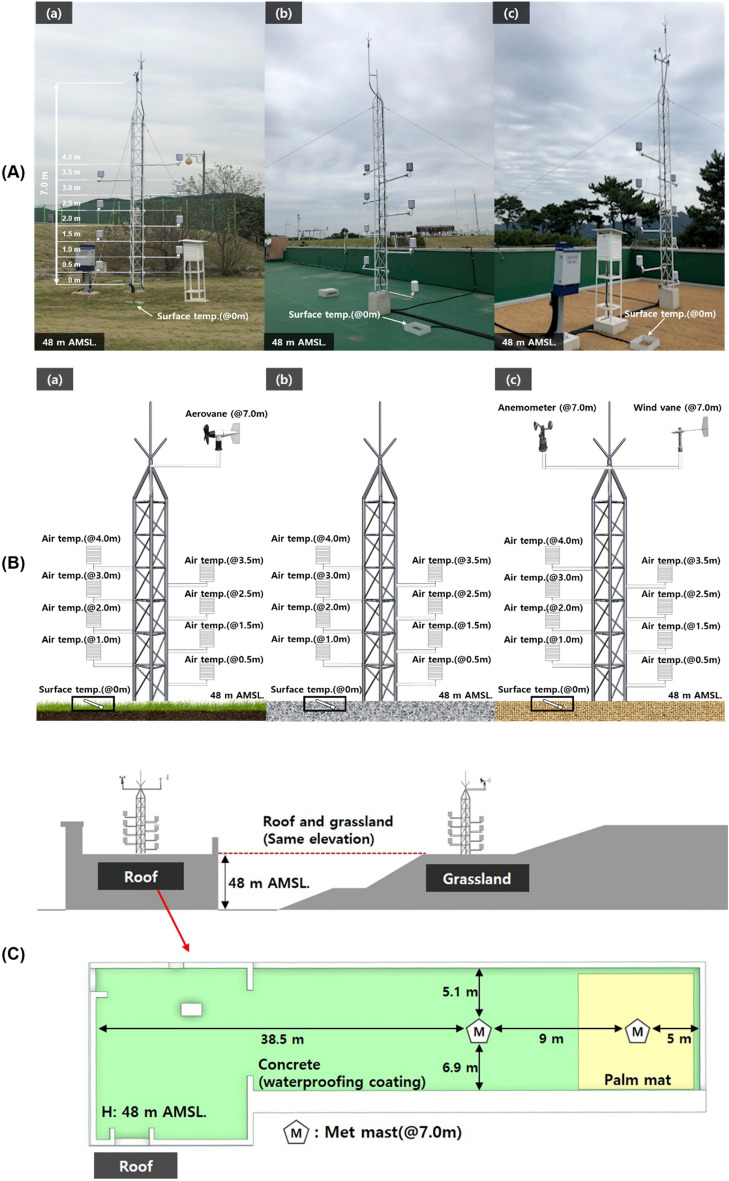
(**A**) Outdoor comparison experiment with different ground material types; (**B**) layout of instrumentation of the different ground materials: grassland (**a**), concrete (**b**), and palm mat (**c**); (**C**) sectional view of the test site. Photograph (**A**) by Byeongtaek Kim. (**B**) was created with SOLIDWORKS Premium version 2021, https://www.solidworks.com/.

To derive the optimal height for measuring the rooftop temperature, the grassland and rooftop (concrete and palm mat) where temperature was observed were configured to be located at the same altitude (48 m; Fig. 2). Palm mat, which was installed to derive the optimal material for rooftop temperature observation, was spread across 10 × 10 m (width × length) according to the KMA weather observation installation guidelines^27^. A four-wire PT100 temperature sensor (JS-RTD100, Jinsung-eng), with a measurement range of − 50 to 100 °C and an accuracy of ± 0.3 °C, was connected to a data logger (CR1000X, Campbell) and used for data collection. The temperature was observed at heights of 0, 0.5, 1.0, 1.5, 2.0, 2.5, 3.0, 3.5, and 4.0 m. In addition, the temperature sensor was installed in an aspirator with a suction fan-type solar radiation shield to minimize the effect of solar radiation. To analyze the temperature change due to the influence of wind speed and wind direction, an aerovane-type (05,103, RMyoung) wind sensor, with wind speed and wind direction accuracies of ± 0.3 m/s and ± 3°, respectively, was installed on the grassland. A three-cup anemometer (JS-WS2082, Jinsung-eng), with a measurement range of 0–75 m/s and an accuracy of ± 0.3 m/s, and a wind vane (JS-WD2081, Jinseong-eng), with an accuracy of ± 0.7°, were installed on the rooftop (Table 1).

**Table 1:** Description of the observation equipment for different environmental parameters.

Parameter	Instrument type	Height (m)	Location
Air temperature	Sensor with four-wire PT 100	0, 0.5, 1.0, 1.5, 2.0, 2.5, 3.0, 3.5, and 4.0	Grassland, concrete, and palm mat
Wind	Aerovane, Three-cup, Wind vane	7	Grassland: Aerovane, Palm mat: Three-cup, Wind vane
Solar radiation	Shield having an aspirator with a suction fan	0.5, 1.0, 1.5, 2.0, 2.5, 3.0, 3.5, and 4.0	Grassland, concrete, and palm mat

### Data processing

The study site was monitored for 1 year (December 2020–November 2021). All data were collected at 1 min intervals. The quality control of the data was conducted by range, step, and persistence tests^36^, after which 70.5% of the total data (370,493 out of 525,600 observations) were used for analysis. Notably, during the observation period, some data were missing due to network failures caused by lightning, cold waves, and hub failures, and one observation was missing due to sensor replacement.

## Results and discussion

### Comparison of temperature differences at the same height from the grassland

**Figure 3:**
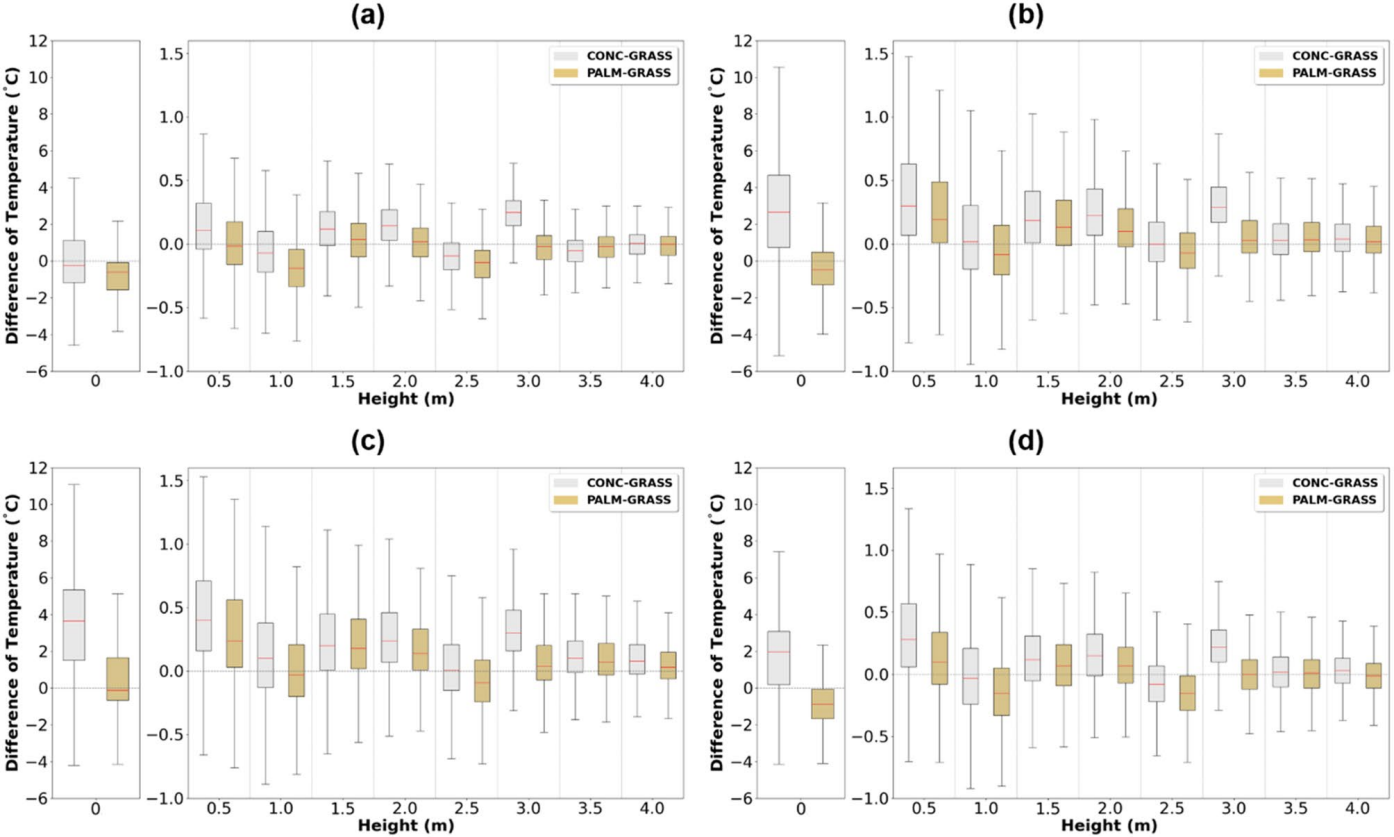
Seasonal characteristics of air temperature difference between concrete–grassland (gray) and palm mat–grassland (brown) at each height for winter (**a**), spring (**b**), summer (**c**), and autumn (**d**).

To determine the altitude at which the rooftop indicator has minimal influence, the temperature difference at the same height was analyzed by season based on the grassland temperature (Fig. 3). Although a marginal seasonal difference was observed, the median ranges of temperature difference were − 0.87 to 0.24 °C and − 0.23 to 3.66 °C from the ground surface (z = 0 m) to 2.5 m height where the palm mat and concrete were installed, respectively. In palm mat and concrete, the median ranges of temperature difference were − 0.87 to − 0.11 °C and − 0.23 to 3.66 °C, respectively. Although the temperature difference during summer was 3.66 °C in concrete, the difference was − 0.11 °C in palm mat. During the observation period, at 3 and 3.5 m above the palm mat and concrete, respectively, the average temperature difference between the palm mat and concrete was 0.02 °C, which was less than that at other heights, whereas the median and interquartile range (IQR) of the temperature difference from the grassland at the same height were 0.01 °C and 0.24 °C, respectively (Table 2). This indicates that the surface heating effect of concrete or palm mat did not change considerably above 3 and 3.5 m height. Contrastingly, the surface heating effect was comparatively stronger at heights closer to the ground surface; moreover, the effect was more pronounced in summer than in winter. The IQR of temperature difference was 1.50 °C and 2.28 °C in winter (Fig. 3a) and 2.32 °C and 3.83 °C in summer (Fig. 3c) for the palm mat and concrete, respectively, based on grassland. Furthermore, the median and IQR of the temperature difference were 0.88 °C and 4.25 °C in palm mat and 2.55 °C and 5.90 °C in concrete, respectively (Table 2). Thus, the effect of surface heating in concrete was higher than that in palm mat.

**Table 2:** Temperature differences at the same height from the grassland for all seasons (PALM: palm mat–grassland, CONC: concrete–grassland).

Height (m)	1st quartile (°C)		Median (°C)		3rd quartile (°C)		IQR (°C)	
	PALM	CONC	PALM	CONC	PALM	CONC	PALM	CONC
0	− 0.62	− 0.07	0.88	2.55	3.63	5.83	4.25	5.9
0.5	− 0.06	0.04	0.11	0.25	0.39	0.56	0.45	0.52
1	− 0.28	− 0.2	− 0.13	− 0.01	0.09	0.25	0.37	0.45
1.5	− 0.05	− 0.01	0.09	0.15	0.29	0.35	0.34	0.36
2	− 0.05	0.04	0.07	0.18	0.24	0.37	0.29	0.33
2.5	− 0.25	− 0.18	− 0.12	− 0.05	0.03	0.11	0.28	0.29
3	− 0.1	0.14	0.01	0.26	0.14	0.4	0.24	0.26
3.5	− 0.08	− 0.09	0.02	0.01	0.14	0.15	0.22	0.24
4	− 0.08	− 0.06	0.01	0.03	0.11	0.14	0.19	0.2

### Comparison of temperature differences at 1.5 m height from the grassland

**Figure 4:**
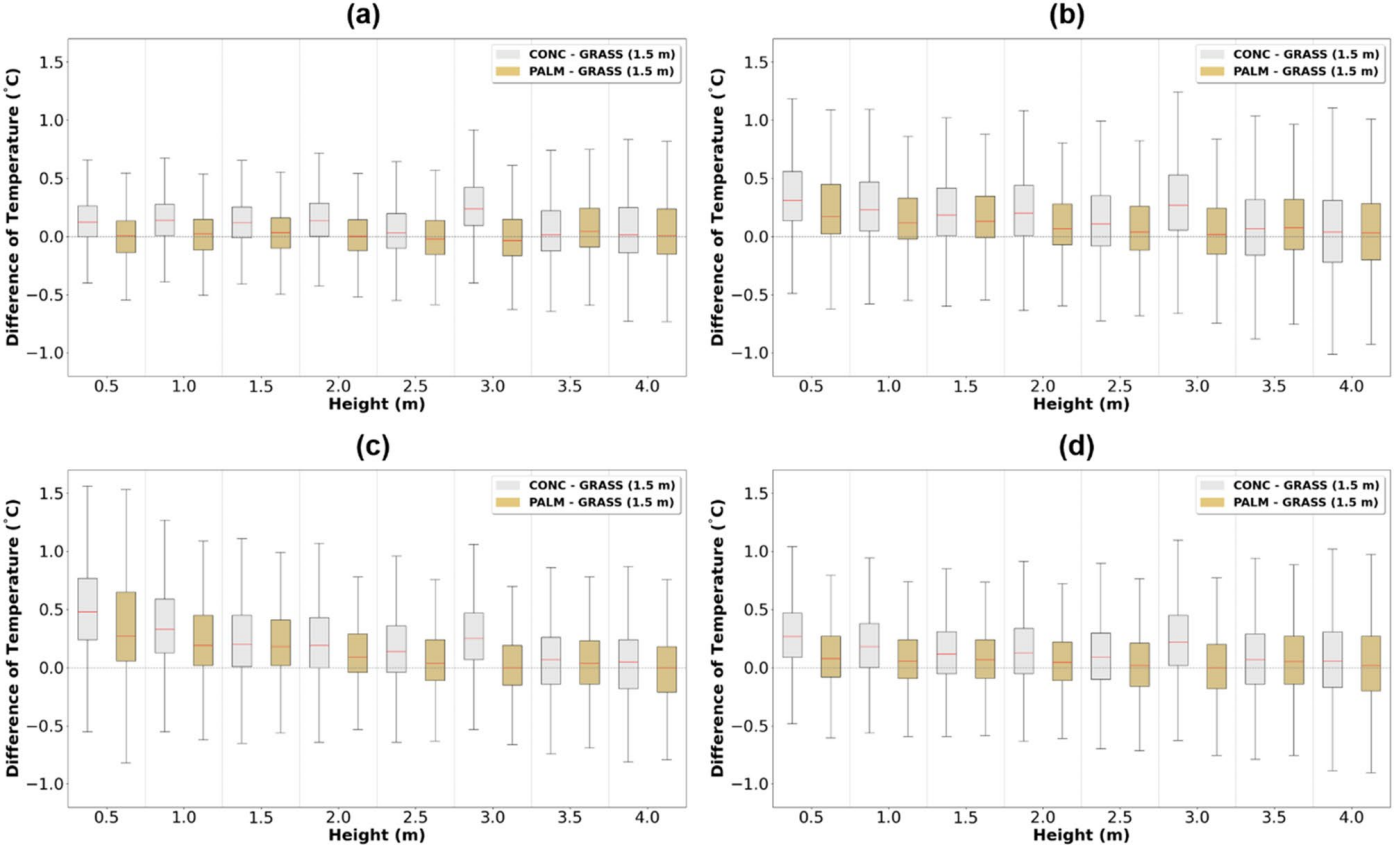
Seasonal characteristics of air temperature difference between concrete–grassland (gray) and palm mat–grassland (brown) at 1.5 m height above the ground for winter (**a**), spring (**b**), summer (**c**), and autumn (**d**).

According to the KMA guidelines, temperature observations should be made at a height of 1.5 m from the grasslands (hereafter referred to as “grasslands-1.5”)^27^. Thus, in this section, analysis was conducted to compare palm mat and concrete temperatures with that of grassland-1.5. The difference between the temperature measured at grassland-1.5 and the temperatures at heights of 0.5, 1.0, 1.5, 2.0, 2.5, 3.0, 3.5, and 4.0 above the concrete and palm mat were analyzed seasonally (Fig. 4). The difference in the median value was the smallest in winter (Fig. 4(a); palm mat: − 0.02 to 0.04 °C, concrete: 0.2–0.24 °C) and largest in summer (Fig. 4c; palm mat: 0.00–0.27 °C, concrete: 0.05–0.48 °C). The heights of the ground weather observatory that showed the smallest difference to grassland-1.5 temperatures were 2.5–3.0 m and 3.5–4.0 m above the palm mat and concrete, respectively. The median temperature values at 2.5 m and 3.0 m above the palm mat were 0.01 °C and − 0.01 °C, respectively, while those at 3.5 m and 4.0 m above the concrete were 0.05 °C and 0.04 °C, respectively (Table 3).

**Table 3:** Temperature differences at 1.5 m height from the grassland for all seasons (PALM: Palm mat–grassland, CONC: Concrete–grassland).

Height (m)	1st quartile (°C)		Median (°C)		3rd quartile (°C)		IQR (°C)	
	PALM	CONC	PALM	CONC	PALM	CONC	PALM	CONC
0.5	− 0.04	0.09	0.11	0.26	0.35	0.52	0.39	0.43
1	− 0.06	0.04	0.08	0.20	0.29	0.42	0.34	0.38
1.5	− 0.05	− 0.01	0.09	0.15	0.29	0.35	0.34	0.36
2	− 0.09	− 0.01	0.05	0.16	0.24	0.37	0.33	0.38
2.5	− 0.14	− 0.08	0.01	0.08	0.21	0.30	0.35	0.38
3	− 0.16	0.06	− 0.01	0.24	0.20	0.47	0.36	0.41
3.5	− 0.12	− 0.14	0.05	0.05	0.27	0.27	0.39	0.41
4	− 0.19	− 0.17	0.02	0.04	0.24	0.28	0.43	0.45

**Figure 5:**
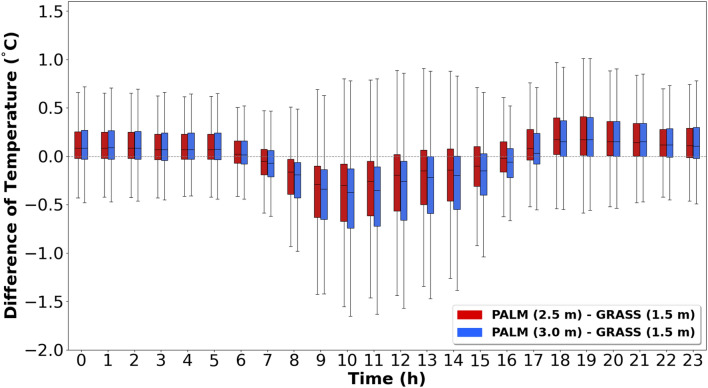
Diurnal temperature differences between the palm mat at 2.5 m (red) and 3.0 m (blue) heights compared with the grassland at 1.5 m (grassland-1.5) temperature at 1.5 m.

**Figure 6:**
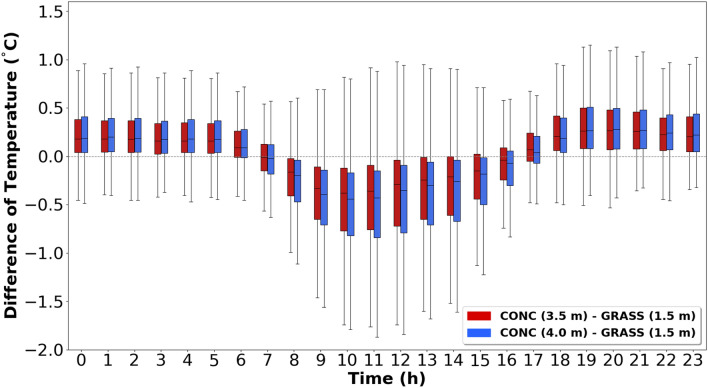
Diurnal temperature differences between the concrete at 3.5 m (red) and 4.0 m (blue) heights compared with the grassland-1.5 temperature.

To determine at which height the palm mat and concrete temperatures were most similar to grassland-1.5, the daily temperature differences were analyzed between the palm mat (at 2.5 m and 3.0 m) and concrete (at 3.5 m and 4.0 m) with reference to grassland-1.5 (Figs. 5 and 6). The median temperature range at 3.0 m above the palm mat was − 0.01 to 0.09 °C higher than that at 2.5 m (Fig. 5), whereas the median temperature range at 3.5 m above the concrete was − 0.02 to 0.07 °C higher than that at 4.0 m (Fig. 6). Thus, the temperatures measured at 2.5 m and 3.5 m heights above the palm mat and concrete (hereafter referred to as “palm mat-2.5” and “concrete-3.5”), respectively, were closer to that measured at grassland-1.5 than at other heights. In addition, the temperatures in the palm mats and concrete were higher and lower than that in the grassland during nighttime and daytime, respectively, and this trend was more prominent in the concrete than in the palm mats. The temperature difference between the grassland and rooftop (concrete and palm mat) was higher in summer than in winter (Fig. 4) because of stronger sunlight intensity in summer that increases the latent heat due to evaporation^37,38^.

**Figure 7:**
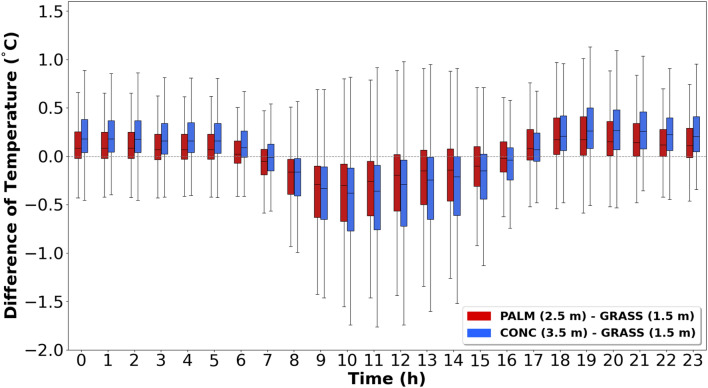
Diurnal variation in the temperature difference in the palm mat (2.5 m) and concrete (3.5 m) considering grassland-1.5. (Red: Palm mat, 2.5 m—grassland, 1.5 m, Blue: Concrete 3.5 m—grassland 1.5 m).

The daily temperature differences at palm mat-2.5 and concrete-3.5 were measured considering grassland-1.5. The median and IQR values were − 0.30 to 0.17 °C and 0.23–0.59 °C, respectively, at palm mat-2.5, and − 0.38 to 0.27 °C and 0.27–0.68 °C, respectively, at concrete-3.5 (Fig. 7). These findings suggested that the temperature measured at palm mat-2.5 was more similar to that measured at grassland-1.5 than that at concrete-3.5.

Thus, installing thermometers on the roof at a height of 2.5 m above palm mat is the best alternative to observe temperatures.

### Relationship of wind speed and rain with temperature difference

**Figure 8:**
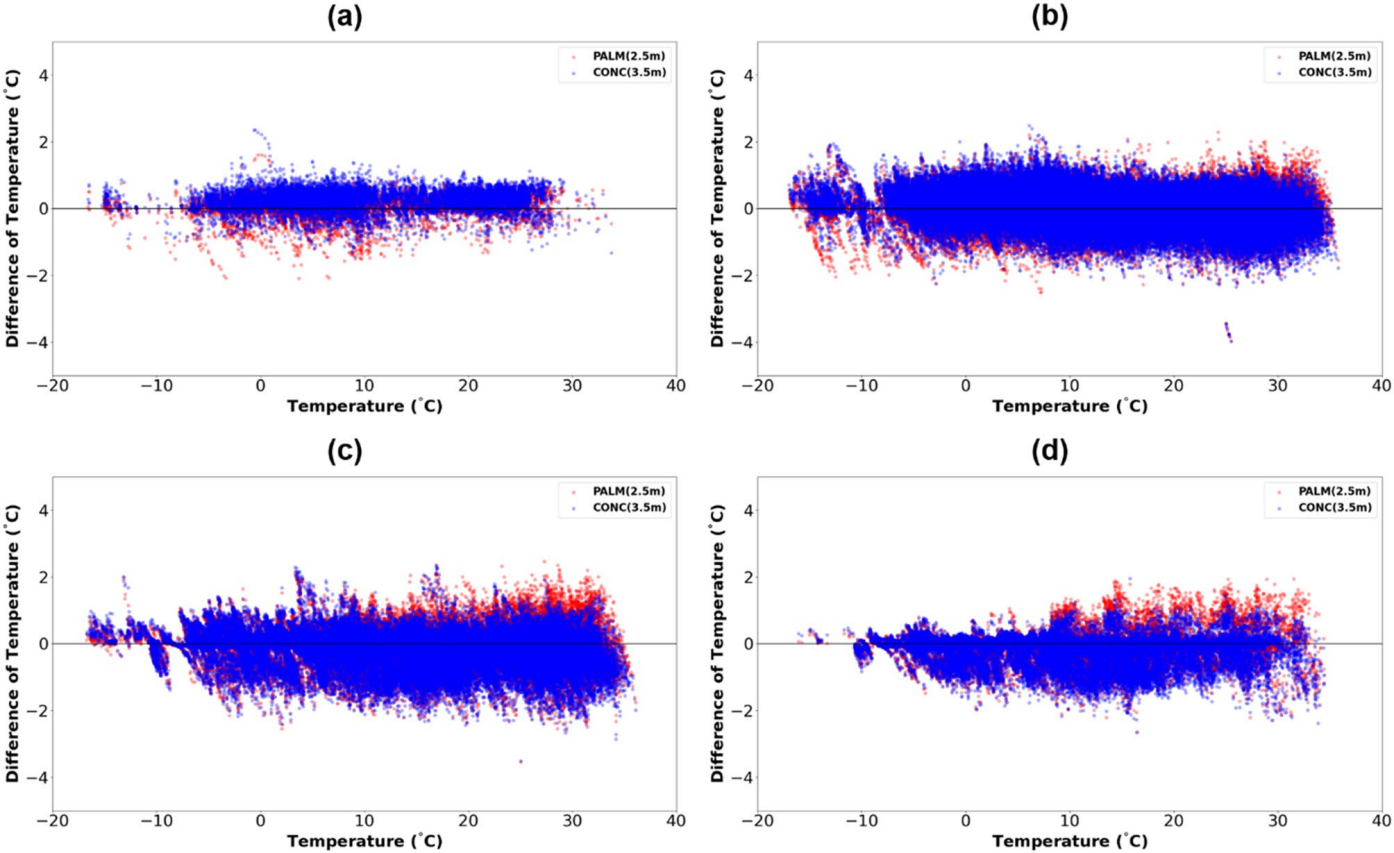
Air temperature difference between the palm mat at 2.5 m (palm mat-2.5) (red) and concrete at 3.5 m (concrete-3.5) (blue) based on the grassland-1.5 temperature- by different wind speed categories, namely, calm wind (**a**), 0–2 m/s (**b**), 2–4 m/s (**c**), and > 4 m/s (**d**).

To analyze the influence of wind speed on the temperature measured in the palm mat-2.5 and concrete-3.5, wind speed was divided into four categories (calm wind, 0–2 m/s, 2–4 m/s, and > 4 m/s) and the temperature difference was analyzed based on the temperature in the grassland-1.5 (Fig. 8). During calm winds, the temperature of the concrete was slightly higher than that of the palm mat (Fig. 8a), possibly because concrete has a higher ground heating effect characterized by increased heat release and air convection than the palm mat and transfers heat to the upper layer without allowing wind-based heat dissipation^39^. Table 4 shows the mean bias error (MBE) and root mean square error (RMSE) of the palm mats and concrete based on the grassland-1.5 for each wind speed category. The temperature difference was positive for calm wind (palm mat: 0.09 °C, concrete: 0.23 °C) and 0–2 m/s (palm mat: 0.05 °C, concrete: 0.10 °C) and negative for 2–4 m/s (palm mat: − 0.06 °C and concrete: − 0.12 °C).

**Table 4:** Statistics of air temperature data by wind speed.

Char	No wind		0–2 m/s		2–4 m/s		> 4 m/s	
	PALM	CONC	PALM	CONC	PALM	CONC	PALM	CONC
MBE (°C)	0.09	0.23	0.05	0.10	− 0.06	− 0.12	− 0.10	− 0.17
RMSE (°C)	0.29	0.38	0.37	0.42	0.47	0.49	0.41	0.44

**Figure 9:**
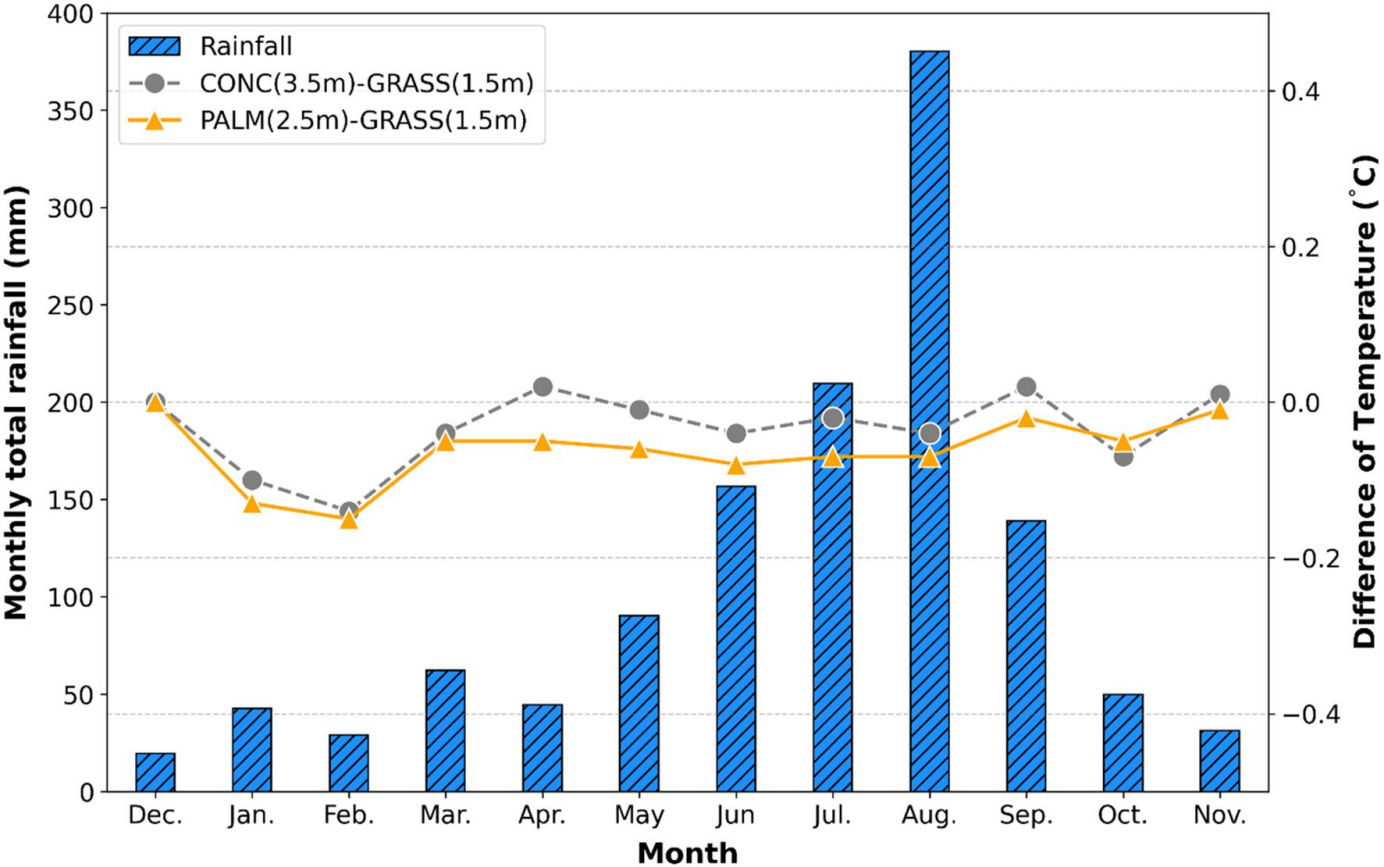
Monthly total rainfall and air temperature difference between the palm mat-2.5 and concrete-3.5 based on the grassland-1.5 temperature by rainfall events.

To analyze the relationship between temperature and precipitation, precipitation data (0.5 mm/h or more) collected at 1-min intervals by ASOS in GSWO were used for analysis. The temperatures of the concrete and palm mat were lower than those of the grassland; furthermore, as precipitation increased, the temperature of the palm mats tended to be lower than that of the concrete (Fig. 9). The RMSE values of the palm mat and concrete were the same, but the MBE value was 0.03 °C lower in concrete than in the palm mat (Table 5). Rainwater is absorbed into the ground of grasslands and may disperse underground, whereas rainwater absorbed in the palm mats installed on the rooftop remains stagnant^40^. Conversely, water is not absorbed easily into concrete. Thus, the temperature of the palm mat was lower than that of the concrete due to the cooling effect of the absorbed precipitation.

**Table 5:** Statistics of air temperature data by rain.

Statistic	PALM (2.5 m)	CONC (3.5 m)
MBE (°C)	− 0.07	− 0.04
RMSE (°C)	0.16	0.16

## Discussion

Surface materials and height are critical factors for measuring temperatures in urban climates. The relevant guidelines state that the installation heights should be 1.25–2.0 m on grassland^1,28–30^. However, with urbanization, it has become very difficult to establish observation environments composed of grassland. In our study, the temperature of different rooftop ground surfaces presented seasonal differences, as indicated by the comparison of the seasonal characteristics of air temperature difference between concrete and palm mat based on grassland (Fig. 3). The temperature difference in winter was obviously lower than that in spring and summer; the temperature difference during summer was 3.66 °C in concrete and − 0.11 °C in palm mat. A similar finding was reported by another study^16^.

Previous studies have shown the relationship between wind speed and precipitation with temperature. The temperature decreases by 0.2 °C on an average every time the wind speed increases by 0.5 m/s; there is no evaporation due to sunlight during the rainy season and the temperature decreases due to cooling^3,41^. As expected, our results (optimal height; concrete 3.5 m, palm mat 2.5 m) are hardly affected by wind and precipitation (See Figs. 8 and 9).

The results of the present work (Fig. 7) show that the optimum surface material and height to measure temperature at the rooftop is palm mat-2.5. These results were analyzed using boxplot (median, quartiles, 1.5 interquartile range). The optimal temperature observation height of 2.5–3.0 m is consistent with that in previous studies^31^, but further study is needed because an insufficient number of sampling data and seasonal characteristics were reflected owing to the wide cycle and short study period. A few previous studies have investigated installation environmental (palm mat and concrete) and optimum height to measure temperature on roofs.

A limitation of the current study was the influence of a building wall (2.5 m) to the east of the rooftop on temperature measurement. The air temperature differences shown in Fig. 3 depict a wall effect characterized by an increase in heat release and air convection and transfer of heat to the upper layer without allowing wind-based heat dissipation. Future studies should focus on the wall effect, which can aid in identifying the trend of air temperature differences using computational fluid dynamics.

## Conclusions

This study derived the optimal height for temperature observation according to the rooftop material (concrete and palm mat) by comparing the temperature data of the rooftop environments and the grassland in GSWO from December 2020 to November 2021. The findings provided observation standards for measuring rooftop temperature according to the rooftop material and the optimal material and observation height for temperature measurement. Accordingly, heights of 2.5 m and 3.5 m above palm mat and concrete, respectively, were determined to be optimum for installing a thermometer on a rooftop for temperature observation. These results can be used to set up optimal temperature monitoring equipment to monitor the occurrences of heat waves, tropical nights phenomenon, and drought in cities considering their specific environmental conditions.

Comparative analysis revealed that the temperature of a concrete rooftop with artificial grass was higher than that of grassland. However, building heights and the surrounding environmental conditions are diverse and complex in real urban environments; therefore, the results of this study may not represent all situations. To this end, future studies should consider the same environmental conditions to evaluate the accuracy of numerical models using historical data and various experimental environments should be developed to represent real urban environments to conduct numerical experiments for accurate temperature measurements in different cities.

## Data Availability

Data are available from the corresponding author upon reasonable request and with the permission of National Institute of Meteorological Sciences (NIMS).

## References

[1] World Meteorological Organization (WMO). Guide to Instruments and Methods of Observation: Volume I Measurement of Meteorological Variables, WMO-No. 8, 2018 ed., 549 (2018). https://library.wmo.int/doc_num.php?explnum_id=10616.

[2] Ren G, Zhou Y, Chu Z, Zhou J, Zhang A, Guo J, Liu X (2008). Urbanization effects on observed surface air temperature trends in North China. J. Clim..

[3] Ngarambe J, Oh JW, Su MA, Santamouris M, Yun GY (2021). Influences of wind speed, sky conditions, land use and land cover characteristics on the magnitude of the urban heat island in Seoul: An exploratory analysis. Sustain. Cities Soc..

[4] He W, Zhang L, Yuan C (2022). Future air temperature projection in high-density tropical cities based on global climate change and urbanization: A study in Singapore. Urban Clim..

[5] Hu Z, Li Y (2022). Effect of urbanization on extreme temperature events in Liaoning Province, China, from a spatiotemporal perspective. Urban Clim..

[6] Tong X, Wang P, Wu S, Luo M (2022). Urbanization effects on high-frequency temperature variability over South China. Urban Clim..

[7] Yao R, Hu Y, Sun P, Bian Y, Liu R, Zhang S (2022). Effects of urbanization on heat waves based on the wet-bulb temperature in the Yangtze River Delta urban agglomeration, China. Urban Clim..

[8] Simon H, Lindén J, Hoffmann D, Braun P, Bruse M, Esper J (2018). Modeling transpiration and leaf temperature of urban trees: A case study evaluating the microclimate model ENVI-met against measurement data. Landsc. Urban Plan..

[9] Yi C, Shin Y, Roh JW (2018). Development of an urban high-resolution air temperature forecast system for local weather information services based on statistical downscaling. Atmosphere.

[10] Detommaso M, Costanzo V, Nocera F (2021). Application of weather data morphing for calibration of urban ENVI-met microclimate models: Results and critical issues. Urban Clim..

[11] Lee JW, Min KH, Lim KS (2022). Comparing 3DVAR and hybrid radar data assimilation methods for heavy rain forecast. Atmos. Res..

[12] Liao Y, Shen X, Zhou J, Ma J, Zhang X, Tang W, Chen Y, Ding L, Wang Z (2022). Surface urban heat island detected by all-weather satellite land surface temperature. Sci. Total Environ..

[13] Aristodemou E, Arcucci R, Mottet L, Robins A, Pain C, Guo YK (2019). Enhancing CFD-LES air pollution prediction accuracy using data assimilation. Build. Environ..

[14] Na HN, Jung WS (2019). A study on improving the prediction accuracy of a Typhoon Disaster prevention model Part I: Sensitivity of the WRF model to high-resolution SST data and meteorological data assimilation. J. Korean Soc. Atmos. Environ..

[15] Tian J, Liu J, Yan D, Ding L, Li C (2019). Ensemble flood forecasting based on a coupled atmospheric-hydrological modeling system with data assimilation. Atmos. Res..

[16] Kuang W, Li Z, Hamdi R (2020). Comparison of surface radiation and turbulent heat fluxes in Olympic Forest Park and on a building roof in Beijing, China. Urban Clim..

[17] Vallati A, Mauri L, Colucci C (2018). Impact of shortwave multiple reflections in an urban street canyon on building thermal energy demands. Energy Build..

[18] Doulos L, Santamouris M, Livada I (2004). Passive cooling of outdoor urban spaces: The role of materials. Sol. Energy.

[19] Synnefa A, Santamouris M, Livada I (2006). A study of the thermal performance of reflective coatings for the urban environment. Sol. Energy..

[20] Loughner CP, Allen DJ, Zhang DL, Pickering KE, Dickerson RR, Landry L (2012). Roles of urban tree canopy and buildings in urban heat island effects: Parameterization and preliminary results. J. Appl. Meteorol. Climatol..

[21] Cascone S, Catania F, Gagliano A, Sciuto G (2018). A comprehensive study on green roof performance for retrofitting existing buildings. Build. Environ..

[22] Yazdani H, Baneshi M (2021). Building energy comparison for dynamic cool roofs and green roofs under various climates. Sol. Energy..

[23] Jaafar H, Lakkis I, Yeretzian A (2022). Impact of boundary conditions in a microclimate model on mitigation strategies affecting temperature, relative humidity, and wind speed in a Mediterranean city. Build. Environ..

[24] Yeo K, Jung Y (2013). An analysis of effect of green roofs in urbanized areas on runoff alleviation and cost estimation. Seoul inst..

[25] Lee BS, Kwon HK, Kim JG, Kim JH (2016). The effect of green roof load on the structural design of roof slab of LH housing and service facilities. LHI J. Hous. Urban Affairs.

[26] Shin EH, Kim HS (2019). Benefit–cost analysis of green roof initiative projects: The case of jung-gu. Seoul. Sustainability.

[27] KMA. Guidelines for installation and operation of meteorological observatory (2019). https://book.kma.go.kr/search/DetailView.ax?sid=1&cid=37744.

[28] AASC (2019). Recommendations and Best Practices for Mesonets, version 1.

[29] EPA. Meteorological monitoring guidance for regulatory modeling applications, p. EPA-454/R-99-005. United States Environmental Protection Agency, Research Triangle Park, North Carolina (2000). https://www.epa.gov/sites/default/files/2020-10/documents/mmgrma_0.pdf.

[30] EPA. Quality assurance handbook for air pollution measurement systems. Vol. IV, Meteorological Measurements. EPA-454/B-08-002. United States Environmental Protection Agency, Research Triangle Park, North Carolina (2008). https://www3.epa.gov/ttnamti1/files/ambient/pm25/qa/Final%20Handbook%20Document%201_17.pdf.

[31] Jung, G. M. A study on the proper height of AWS temperature sensor on the roof, Weather work improvement announcement book, KMA, pp. 77–88 (1994). https://book.kma.go.kr/viewer/MediaViewer.ax?cid=11512&rid=5&moi=2787.

[32] Kim, G. B., Lee, S. G., Lim, J. S., Chio, K. M., Gwon, D. S., Jung, H. H., Kim, H. S., Shin, Y. S. A study on the radiation effects of AWS installed on the roof, Gangwon weather characteristics book. **8**, 107–113 (1999). https://book.kma.go.kr/viewer/MediaViewer.ax?cid=19986&rid=5&moi=2620.

[33] Joo HD, Lee MJ, Ham IW (2005). The characteristics of air temperature according to the location of automatic weather system. J. Atmos..

[34] NIMS (2021). Annual Report of the Standard Weather Observatories in Korea.

[35] KMA. A detailed analysis of climate change in Gochang-gun, North Jeolla province, KMA, p. 92 (2017). http://www.climate.go.kr/home/cc_data/2018/scenario/analysis_report/Jeollabukdo_Gochanggun.pdf.

[36] Estévez J, Gavilán P, Giráldez JV (2011). Guidelines on validation procedures for meteorological data from automatic weather stations. J. Hydrol..

[37] Sharma A, Conry P, Fernando HJS, Hamlet AF, Hellmann JJ, Chen F (2016). Green and cool roofs to mitigate urban heat island effects in the Chicago metropolitan area: Evaluation with a regional climate model. Environ. Res. Lett..

[38] Zou Z, Yan C, Yu L, Jiang X, Ding J, Qin L, Wang B, Qiu G (2021). Impacts of land use/land cover types on interactions between urban heat island effects and heat waves. Build. Environ..

[39] Zingre KT, Wan MP, Tong S, Li H, Chang VW-C, Wong SK, Thian Toh WB, Leng Lee IY (2015). Modeling of cool roof heat transfer in tropical climate. Renew. Energy..

[40] Yun SH, Kim ES, Piao ZG, Jeon YH, Kang HW, Kim SH, Kim JY, Kang HM, Ham EK, Lee DK (2021). The effect of temperature reduction of green roof using rainwater storage tank. J. Korean Environ. Res. Tech..

[41] Trenberth KE, Shea DJ (2005). Relationships between precipitation and surface temperature. Geophys. Res. Lett..

